# Intralocus Sexual Conflict Diminishes the Benefits of Sexual Selection

**DOI:** 10.1371/journal.pbio.0040356

**Published:** 2006-10-24

**Authors:** Alison Pischedda, Adam K Chippindale

**Affiliations:** Department of Biology, Queen's University, Kingston, Ontario, Canada; Duke University, United States of America

## Abstract

Evolution based on the benefits of acquiring “good genes” in sexual selection is only plausible with the reliable transmission of genetic quality from one generation to the next. Accumulating evidence suggests that sexually antagonistic (SA) genes with opposite effects on Darwinian fitness when expressed in the two different sexes may be common in animals and plants. These SA genes should weaken the potential indirect genetic benefits of sexual selection by reducing the fitness of opposite-sex progeny from high-fitness parents. Here we use hemiclonal analysis in the fruit fly, *Drosophila melanogaster,* to directly measure the inheritance of fitness across generations, over the entire genome. We show that any potential genetic benefits of sexual selection in this system are not merely weakened, but completely reversed over one generation because high-fitness males produce low-fitness daughters and high-fitness mothers produce low-fitness sons. Moreover, male fitness was not inherited by sons, consistent with both theory and recent evidence connecting this form of SA variation with the X chromosome. This inheritance pattern may help to explain how genetic variation for fitness is sustained despite strong sexual selection, and why the ZW sex chromosome system found in birds and butterflies appears to foster the evolution of extreme secondary sexual characters in males.

## Abstract 2

Hemiclonal analysis of*Drosophila melanogaster* reveals that high-fitness males produce low-fitness daughters and high-fitness mothers produce low-fitness sons, with implications for models of sexual selection.

## Introduction

Sexual conflict, which arises whenever males and females have different reproductive interests, takes on two fundamentally different forms genetically. Interlocus sexual conflict involves direct sexual interactions and has become particularly topical [1,2] because of its potential to generate co-evolutionary arms races between the sexes [1,3] that may contribute to rapid evolution and speciation [4–6]. While interlocus conflict involves different genes in each sex, intralocus sexual conflict creates a tug-of-war between the sexes because the same allelic variation has opposite effects on Darwinian fitness when expressed in each sex. Intralocus conflict may be a transient phase that is resolved by sex-limited gene expression [7], genomic imprinting [8], or reduced opposite-sex heritabilities [9], each of which may result in sexual dimorphism by restricting a gene's expression to only the sex that it benefits. However, recent studies have emphasized that substantial intralocus conflict can remain unresolved in the genome [10–14], thereby reducing the average fitness of each sex. Thus, among the implications of this form of conflict are two central problems of evolutionary genetics: the costs of sexual reproduction and the maintenance of genetic variation for fitness in the face of selection [15,16].

In the *Drosophila* model system, evidence for intralocus sexual conflict has come from both selection experiments [17,18] and hemiclonal analysis in which identical haploid genomes were expressed in both males and females whose relative fitness was measured [10]. Although the latter approach revealed a strong positive genetic correlation for juvenile survival, a stage in which the sexes look and behave similarly, adult reproductive success yielded a strong negative genetic correlation between the sexes [10]. These data suggested that intralocus sexual conflict is only manifested when the two sexes have markedly different phenotypes, and that the average individual was expressing a substantial load of sexually antagonistic (SA) variation. Moreover, subsequent work found that SA variation was especially abundant on the X chromosome [16] as predicted by theory [15], with the X chromosome explaining a large fraction (estimated 97%) of the SA effects observed for the whole genome [16].

One predicted consequence of intralocus SA variation is an inverted pattern of fitness inheritance from mothers to sons and fathers to daughters, potentially interfering with sexual selection for good genes. This effect would be exaggerated by X-linkage of SA genes because males only transmit their X chromosomes to daughters. As a result, the indirect benefits of choosing high-fitness males would be diminished because daughters would experience reduced fitness and sons would not benefit from paternal fitness. Here, we test these predictions in D. melanogaster by experimentally mating female and male flies of high and low genetic quality and determining the fitness of their offspring.

## Results/Discussion

We obtained high- and low-fitness parents using hemiclonal analysis [10], which allows a nearly complete haploid set of chromosomes (99.5% of the genome, consisting of all major chromosomes except the tiny fourth chromosome) to be randomly sampled, cloned, and then tested in multiple random genetic backgrounds. From independent surveys of fitness variation in females and males (see Materials and Methods), three lines with the highest and lowest competitive reproductive success for each sex (paternity success for males and egg production for females) were selected as high- and low-fitness parents for further study. The high-fitness female lines produced 35% more eggs than low-fitness females (*t* = 4.51, *df* = 4, *p* = 0.0108; Figure 1), whereas high-fitness male lines sired 44% more offspring than low-fitness males (*t* = 11.31, *df* = 4, *p* = 0.0003; Figure 1).

**Figure 1:**
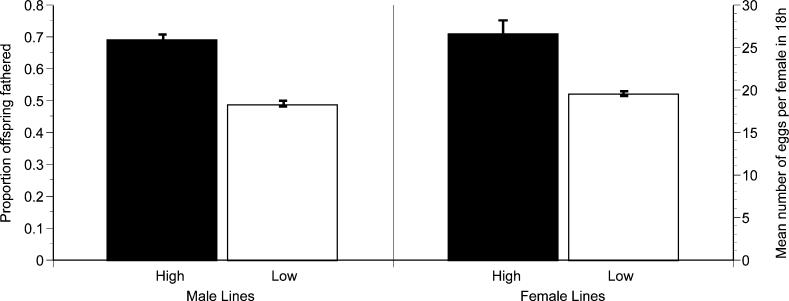
Differences in Fitness between the Lines Selected as Parents for Experimental Crosses Were Substantial and Genetically Mediated In the male fitness survey (*n* = 70), the mean proportion of offspring fathered (± 95% confidence interval [CI]) was 0.544 ± 0.016. In the female fitness survey (*n* = 12), the mean fecundity was 22.85 ± 1.87. Error bars indicate standard errors.

To measure the inheritance pattern for fitness, we obtained offspring from experimental matings between high- and low-fitness male and female lines in all possible combinations (36 crosses in total). Offspring viability was used to test for maternal effects and major mutants, under the assumption that variation influencing organismal function common to both sexes would be expressed during development and affect both sexes negatively. We found that offspring viability was not significantly influenced by the fitness rating (high or low) of either parent (maternal fitness: *F*_1,24_ = 0.001, *p* = 0.97; paternal fitness: *F*_1,24_ = 0.022, *p* = 0.88), suggesting that maternal effects were weak or absent, and levels of unconditional fitness variation were low. For these reasons, and because total offspring counts (both males and females) were used to estimate viability, we considered only the reproductive success of sons (paternity success) and daughters (egg production) for our offspring fitness estimates. However, none of our findings were affected if we incorporated viability (i.e., assuming a 1:1 sex ratio from viability counts) into these fitness estimates.

We found that maternal fitness strongly affected the adult fitness of both daughters (Figure 2A) and sons (Figure 2B), but in opposite directions. High-fitness mothers produced daughters that were, on average, 7% more fit than daughters produced by low-fitness mothers (Table 1). In contrast, high-fitness mothers produced sons that were substantially less successful (11%) than low-fitness mothers (Table 1). Similarly, daughters sired by high-fitness males were, on average, almost 7% less fit than daughters sired by low-fitness males (Figure 2A; Table 1). These results are consistent with the effects of intralocus SA fitness variation. The fact that paternal fitness had no significant effect on the fitness of sons (Figure 2B; Table 1) is also consistent with the inheritance of X-linked SA fitness variation, because only the X-inheriting sex (daughters) should be affected by paternal fitness. These inheritance patterns are supported by one-tailed Spearman rank correlation coefficients: maternal fitness was positively correlated with daughter fitness (*r*_s_ = 0.886, *p* = 0.03) and negatively correlated with son fitness (*r*_s_ = −0.829, *p* = 0.05), whereas paternal fitness correlated negatively with daughter fitness (*r*_s_ = −0.886, *p* = 0.03), but was unrelated to son fitness (*r*_s_ = 0.371, *p* > 0.05). Considering that there were 24 possible outcomes for the rank ordering of fitness in both the son and the daughter experiments, the fact that the pattern predicted by sex-linked SA genes was realized in each (binomial probability of *p* = 0.04 for each assay), lends further (nonparametric) support to these conclusions.

**Figure 2:**
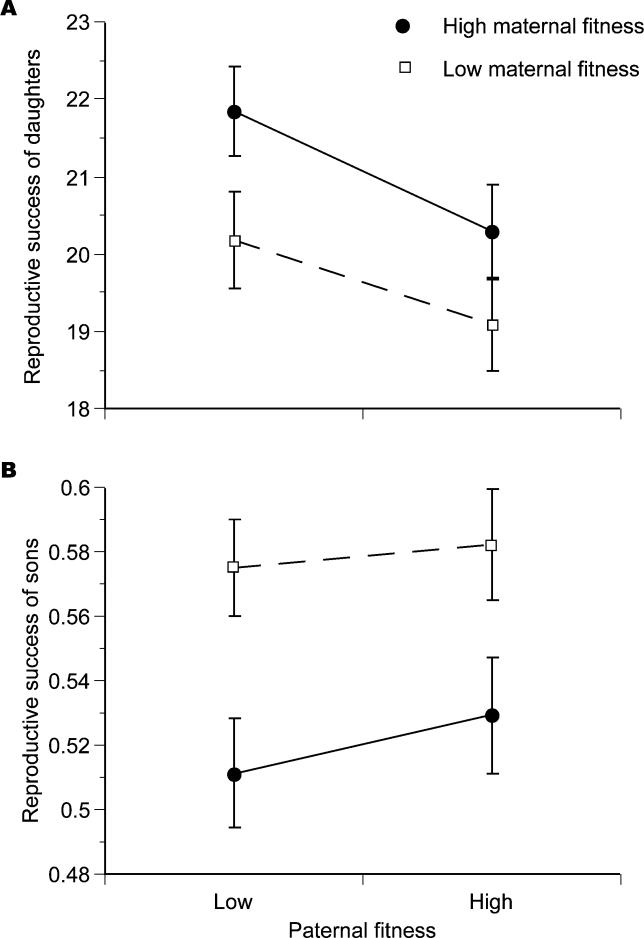
X-Linked SA Variation Results in an Inverted Pattern of Fitness Inheritance from Fathers to Daughters and Mothers to Sons (A) Daughter reproductive success, measured as egg production in an 18-h period, was positively related to maternal fitness and negatively related to paternal fitness. (B) In contrast, son reproductive success, measured as the proportion of offspring fathered, was negatively related to maternal fitness and unaffected by paternal fitness. Error bars indicate standard errors.

**Table 1:**
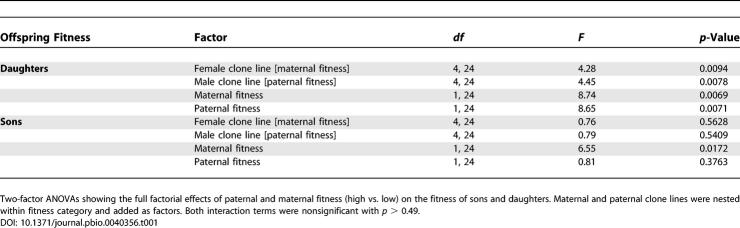
The Effects of Maternal and Paternal Fitness on Offspring Fitness

If these patterns are typical, then our results have counterintuitive implications for models of sexual selection via either male–male competition or female mate choice. Females whose mates have high mating success will gain no indirect benefits through sons (or their offspring) and pay a cost in the reduced fitness of daughters. Although grandsons produced by such females may recover some fitness loss incurred in the F_1_ generation, these will be weakened by recombination, a full generation delay, and reduced fitness of granddaughters. Therefore, although compensation in the F_2_ generation is possible, it is predicted to be slight. These patterns of indirect effects are in direct conflict with the “good genes” and “sexy sons” theories of sexual selection [19]. Even with genetic monogamy, if pairs form through positive assortative mating for fitness, the highest fitness male and female genotypes in a population will yield only medium-fitness daughters and low-fitness sons (Figure 2). In fact, when the fitness means for both sons and daughters from each combination are expressed as relative values (proportion of the highest measured fitness) and averaged, we find that high-fitness males and females have the lowest overall offspring fitness (0.919) of any combination. Instead, it is the combination of low-fitness females with low-fitness males that produces the highest averaged offspring fitness (0.956), with the other combinations producing intermediate-fitness offspring (low female × high male = 0.937; high female × low male = 0.939). In other words, fitness shows regression towards the mean value in response to positive assortative mating or any form of sexual selection on males. These findings may help to explain the maintenance of genetic diversity for fitness, even in the face of strong sexual selection (e.g., the lek paradox; ref [20]).

Our data also have important implications for sexual selection in organisms with different sex chromosome systems. The ZW/ZZ sexual system present in bird and butterfly species has long been linked to more elaborate male secondary sexual traits and displays [21–24], a pattern quantified by Reeve and Pfennig [22]. We suggest that sexual selection is disrupted in XX/XY systems by sex-linked SA variation because males are unable to transmit X-linked preferred traits to their sons. In contrast, males in ZW/ZZ systems are homogametic (ZZ), and sexual selection on males carrying sex-linked SA variation may be more efficient because sons directly inherit the paternal Z chromosome. In addition, a recent theoretical model of sexual selection found that female preferences are more likely to drive the accumulation of male-benefit sex-linked SA traits in ZW systems [24]. Because paternal fitness affects the fitness of both sons and daughters, and SA variation in the homogametic sex is predicted to be dominant in expression [15,16], females in ZW systems may be under particularly strong selection to adjust the sex ratio of their offspring based on the quality of their mates, as has been shown in some bird species [25,26]. If SA variation is taxonomically widespread, then this combination of factors predicts stronger sexual selection via male display and female mate choice in ZW systems such as birds.

These and prior data from *Drosophila* laboratory populations [7,10,16] suggest that intralocus sexual conflict may be an important factor in maintaining genetic variation for fitness in populations. It is important, however, to acknowledge potential specificities of the system. First, D. melanogaster has a relatively large X chromosome (approximately 20% of the genome; ref [27]), which may make the form of SA variation documented here more prevalent than in other species. Second, the use of laboratory populations to study intralocus conflict has been criticized on the grounds that a history of selection for a relatively constant environment may remove naturally selected fitness variation, exaggerating the importance of SA genes [28]. However, the same points can be used to argue that laboratory populations of fruit flies are ideal for identifying SA variation. The removal of generally maladapted genotypes will serve to highlight the differences generated by disparate fitness strategies between the sexes, allowing us to estimate the magnitude of intralocus sexual conflict and predict its consequences under more variable conditions, in which positive intersexual genetic correlations are likely to be stronger.

Although intralocus sexual conflict is difficult to measure in the field, several recent studies have reported analogous patterns to our own in natural populations. For example, in the cricket *Allonemobius socius,* a male's field-determined mating success was strongly negatively correlated with his daughter's reproductive success [14], and field studies with side-blotched lizards found that dominant polygynous males produced daughters with decreased viability [11]. Although neither of these studies directly measured fitness, or investigated the influence of sex chromosomes or maternal condition, these patterns are congruent with our experimental results in *Drosophila.* Thus, our results contribute to a growing body of evidence and theory supporting a significant role for SA genes in shaping patterns of fitness inheritance.

Intralocus sexual conflict will result whenever the two sexes are under disruptive selection acting upon a shared character, but are genetically constrained from evolving in different directions (i.e., becoming sexually dimorphic). Many genes affecting the sexual phenotype are likely to be subject to this pattern of selection and constraint, and sex chromosomes appear to support substantial polymorphism for SA alleles [15,16]. We therefore suggest that intralocus sexual conflict is a significant force that is likely to be widely taxonomically distributed across sexual species. Since this form of conflict reduces Darwinian fitness by undermining fertility, it may help to explain the paradoxical maintenance of genetic variation for fitness in the face of selection. By extension, intralocus sexual conflict is likely to be implicated in generating the spectrum of sexual preferences and behaviors seen in sexual animals, as well as variation in their physiological underpinnings.

## Materials and Methods

### Creation of low- and high-fitness male and female hemiclones.

The protocols for sampling, cytogenetically cloning, and then expressing Chromosome I(X), II, and III haplotypes as “hemiclones” are described in detail elsewhere [10]. Briefly, specially constructed clone-generator female lines are used to enforce co-segregation and father–son transmission of all three major chromosomes, facilitated by the lack of molecular recombination in Dipteran males. By crossing these males to females with the appropriate karyotype [10], wild-type sons or daughters may be produced, each carrying the focal haplotype paired with a random sampling of chromosomes from the original base population.

For the male fitness survey, 70 haplotypes were randomly drawn from a large base population (LH_M_; ref [10]), amplified into several hundred copies, and then expressed by males containing random genetic backgrounds from the same base population (LH_M_). Male reproductive success was measured by combining five hemiclone males with ten competitor males taken from a replica of the base population carrying the recessive brown-eyed *bw* mutation (LH_M_-*bw*). These males competed for matings with 15 virgin LH_M_-*bw* females in vials containing food medium and 10 mg of yeast for 2.5 d. At this time, ten LH_M_-*bw* females were isolated from each vial and individually transferred into test tubes containing food medium to oviposit for 16 h. Male reproductive success was measured as the total proportion of offspring fathered by the five hemiclone males across the ten females. Significant additive genetic variation in fitness was recorded (random effect analysis of variance [ANOVA]: *F*_69,206_ = 1.69, *p* = 0.0026; estimated *h^2^* of 0.294 from variance component estimate). From this survey of variation, three male clone lines with repeatably low or high reproductive success as males were selected. Female fitness of these hemiclones, assessed in an independent assay, was also considered in selecting the male lines, and although fitness was not significantly correlated between the sexes, these lines were believed to carry SA variation.

For the female fitness survey, a smaller and separate random sample of 12 haplotypes was taken from the LH_M_-*bw* genetic replica of the base population. Hemiclone females were collected as virgins and combined with wild-type males at a density of 16 pairs of flies per vial, corresponding to their normal culture density. Females were housed with these males on food medium and 10 mg of yeast per vial for 2.5 d, at which time each vial was divided into three fresh, unyeasted vials containing four pairs of flies in each. The competitive reproductive success of females expressing these hemiclones was measured as the average number of eggs produced per female in the 18-h period following this, corresponding to their normal selection protocol.

These 12 lines were originally created for mate choice analysis (hence the small survey and recessive marker), but displayed substantial genetic variation in reproductive success (random effect ANOVA: *F*_11,285_ = 8.86, *p* < 0.0001; estimated *h^2^* of 0.53 from variance component estimate). The three female lines with the highest and lowest reproductive success were selected for this study. We are mindful that they are likely to be less differentiated than would be possible with a more extensive survey, as performed in males, but heritability of female fitness is also typically higher in this system [10].

### Generating offspring from male and female hemiclones.

The selected lines were used to generate hemiclone males and females as described above. Males from each of the six male lines were mass-mated to females from each of the six female lines in all combinations, creating 36 types of experimental offspring, all of which were wild type in phenotype (heterozygous for the recessive *bw* allele). Offspring fitness was measured in the same manner as parental fitness, with all experiments designed to closely mimic the culture conditions of our base population. Both parental and offspring fitness experiments were conducted under identical environmental conditions, on standard agar-cornmeal-molasses medium, and housed in humidity-controlled incubators under a 12-h light/12-h dark diurnal cycle at 25 °C.

### Measuring daughter fitness.

From each of the 36 male–female combinations (each replicated four times), 90 eggs from the focal combination and 90 eggs from a competitor stock (the LH_M_-*bw* replica base population) were transferred into vials containing food medium. Viability was measured 11 d following oviposition as the proportion of eggs surviving to adult eclosion. From each juvenile competition vial, two adult competition vials containing fresh medium and a limiting quantity (8 mg) of live yeast were created with 12 LH_M_-*bw* males, six LH_M_-*bw* females, and six experimental females (distinguishable by their red eyes). Although the experimental design used fewer flies (12 pairs) than the standard culture protocol (16 pairs), an equal sex ratio was maintained and the amount of yeast was adjusted accordingly. 2.5 d later, the six experimental females were transferred into fresh, unyeasted food vials with six LH_M_-*bw* males and were allowed to oviposit for 18 h. The reproductive success of daughters was measured as the average number of eggs produced per female in this period. Thus, for each of the 36 male–female combinations, the fertility of 48 daughters was measured in eight groups of six females.

### Measuring son fitness.

Male juvenile competition vials were seeded with eggs as per the female fitness assay. Each set of 36 combinations was replicated five times. In this assay the competitor was a replica of the base population carrying the recessive *st* mutation (LH_M_-*st*), which confers a scarlet-eyed phenotype. Viability was measured on day 11, and flies from each juvenile competition vial were then lightly anaesthetized using CO_2_ and transferred into half-pint bottles containing food medium and 40 mg of yeast. This simulated routine culture, during which flies are anaesthetized and the culture densities are reduced. Transferring the flies into larger bottles allowed the naturally occurring numbers to be preserved at approximately normal densities. Then 2.5 d later, 20 LH_M_-*st* females were isolated from each bottle and individually transferred into test tubes containing food medium to oviposit for 16 h. The reproductive success of sons was measured as the total proportion of offspring fathered across the 20 females, adjusted by the number of males present to account for differences in viability and sex ratios. The use of the LH_M_-*st* competitor stock allowed paternity to be easily assigned, as *st* produces a red-eyed phenotype when expressed with either the *bw* mutation or a wild-type copy of the gene. As a result, LH_M_-*st* females who mated with experimental sons produced red-eyed offspring, whereas those who mated with competitor males produced scarlet-eyed offspring. Because the fertility of the LH_M_-*st* females would be affected by differences in viability or sex ratios in the competition chamber (e.g., higher numbers of females may produce greater competition for limited food resources), we used the proportion of red-eyed (hemiclone) offspring rather than the raw number of offspring to estimate male reproductive success. For each of the 36 male–female combinations, 100 broods were scored to measure male reproductive success.

### Statistical analysis.

Two-factor nested ANOVA was used for analysis of offspring fitness. Full factorial analysis was performed with the main factors “Maternal Fitness” and “Paternal Fitness” where fitness was coded as high or low. The three independent clone lines within each category were also added as nested factors to account for variation between clone lines within each group. Only grand mean values of each population cross were used in the analysis. Because there were no significant interaction terms (all *p* > 0.49), only the main effects were reported. All analyses were performed using JMP 5.0.1 (http://www.jmp.com/).

## Abbreviations

ANOVA: analysis of variance
SA: sexually antagonistic
